# Science and evolution

**DOI:** 10.1590/1678-4685-GMB-2018-0086

**Published:** 2019-02-28

**Authors:** Claudia A.M. Russo, Thiago André

**Affiliations:** 1 Universidade Federal do Rio de Janeiro Universidade Federal do Rio de Janeiro Departamento de Genética Rio de JaneiroRJ Brazil Departamento de Genética, Universidade Federal do Rio de Janeiro, Rio de Janeiro, RJ, Brazil; 2 Universidade Federal do Oeste do Pará Universidade Federal do Oeste do Pará Programa de Pós-Graduação em Biodiversidade SantarémPA Brazil Programa de Pós-Graduação em Biodiversidade, Universidade Federal do Oeste do Pará, Santarém, PA, Brazil

**Keywords:** evolutionary theory, science, scientific method, scientific theory, macroevolution

## Abstract

Evolution is both a fact and a theory. Evolution is widely observable in laboratory and natural populations as they change over time. The fact that we need annual flu vaccines is one example of observable evolution. At the same time, evolutionary theory explains more than observations, as the succession on the fossil record. Hence, evolution is also the scientific theory that embodies biology, including all organisms and their characteristics. In this paper, we emphasize why evolution is the most important theory in biology. Evolution explains every biological detail, similar to how history explains many aspects of a current political situation. Only evolution explains the patterns observed in the fossil record. Examples include the succession in the fossil record; we cannot find the easily fossilized mammals before 300 million years ago; after the extinction of the dinosaurs, the fossil record indicates that mammals and birds radiated throughout the planet. Additionally, the fact that we are able to construct fairly consistent phylogenetic trees using distinct genetic markers in the genome is only explained by evolutionary theory. Finally, we show that the processes that drive evolution, both on short and long time scales, are observable facts.

In recent years, the teaching of creationism within science curricula has become a subject of public debate worldwide (Miller *et al.*, 2006; Reiss 2011). Most of the attention has been given to cases in the United States of America (Jackson *et al.*, 1995; Berkman and Plutzer, 2011; Baltzley, 2016; Ross, 2017), where many bills have been submitted to the Houses of Representatives encouraging teachers to express their criticism about evolution. In more serious cases, such as Turkey, evolution has recently been removed from the high school curriculum (Kingsley, 2017), and in Brazil, intelligent design research has recently reached university level (Silva, 2017). The rise of “anti-vaxxers” and “flat-earthers” openly demonstrates that the anti-science movement is not confined to biology, with devastating consequences such as the vaccine-preventable outbreaks (Miller *et al.*, 2015). At the same time, the anti-science debates have been usually promoted by anti-scientists and have stayed marginal to scientific literature. This explains the rising trend and confirms the need for scientists to hastily step into the scene. With this in mind, we felt compelled to address basic aspects of science and of the scientific method in the evolution versus divine creation debate in a scientific journal.

Science can be defined as being both the criterion for gathering scientific data (scientific method), as well as the explanatory theories that were developed following its criteria (scientific knowledge) (Project 2061 American Association for the Advancement of Science, 1993; Roberts 2007). A few centuries ago, scientists decided to select a small part of human knowledge to restrict the method used to assemble this knowledge. The use of the scientific method does not mean that this is more valuable than other types of knowledge; it is just more reliable in uncovering natural laws (Atkins, 1995).

One should regard science as a process in which scientists formulate hypotheses to explain certain facts and to test their predictive models by confronting their predictions with new facts (Gilbert, 1991). A fact is something that we observe. For instance, when we drop an object, it falls to the ground. This is a fact. The scientific theory that explains why objects fall is the *theory of gravity*. A valid scientific theory can never become a fact (Gould, 1981), as there is always the possibility that a future explanation will better match newly discovered facts.

## Evolution as a fact and theory

Evolution is a population concept. An individual does not evolve; only populations evolve in the face of the genetic changes accumulated from one generation to the next. The flu virus evolves. This explains why last years’ flu vaccine does not work on the current strain of the virus: only the resistant strains of the virus survived last year’s vaccine application. This is a textbook example of evolution by natural selection. Genetic modifications are encountered in the resistant strains; thus, evolution is a fact (Gould, 1981). Mutation, migration, natural selection, and genetic drift are the evolutionary forces that drive genetic changes of natural populations from one generation to the next. This is known among biologists as microevolution.

**Figure 1:**
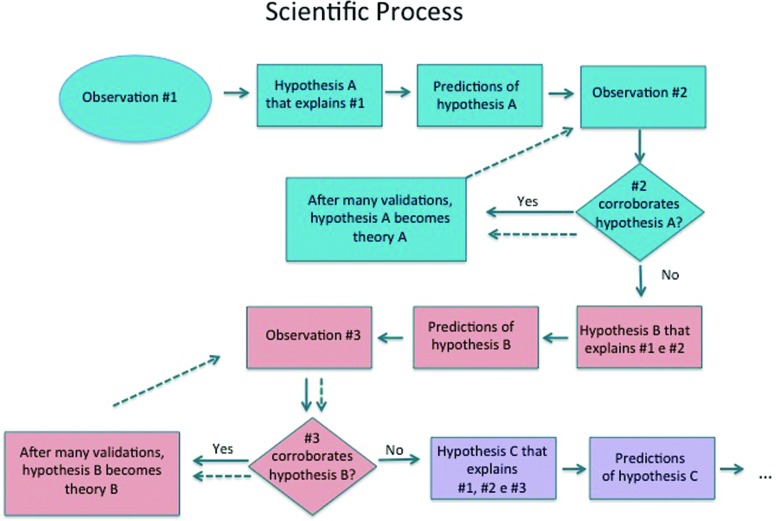
The flow chart illustrates the difference between a scientific hypothesis and a scientific theory. A theory is the highest place an explanation for facts may reach in science.

On the other hand, evolutionary theory explains more than those facts that we can routinely observe. This makes it a theory, but is it *just* a theory? The word *theory* has distinct meanings in science and in lay language (Ghose, 2013). A scientific theory is the utmost position an idea may reach in science. Outside of academia, however, a theory is equivalent to a hypothesis, an idea that explains facts but has never been tested (Futuyama and Kirkpatrick, 2017). This occurs because there seems to be no need for a distinction between hypothesis and theory outside the scope of science. In science, however, this distinction is fundamental. An idea remains a hypothesis if it has never been confronted with new (independently collected) scientific data that would serve as a test for its predictions. If a hypothesis has endured further testing by subsequent scientific experiments, in time it becomes a valid scientific theory (Figure 1).

For any given valid scientific theory there are three possibilities. The first possibility is that the *true* explanation for the facts is entirely different from the valid scientific theory. In this case, all scientific experiments aimed to test the theory were flawed in design or in the interpretation of the results. The second possibility is that the *true* explanation for the facts is more restricted than the current scientific theory claims. In this case, the predictions of the theory agreed with newly collected data because all tests focused on a single (and *true*) aspect of the theory. Finally, the last alternative is that the *true* explanation for the facts is the scientific theory. Science has the tools to reject (first alternative) and to refine (second alternative) scientific theories when they are confronted with new data. However, even theories that endure many tests must still face these three possibilities, as, even in light of the *true* explanation, science does not furnish us the tools to perceive truthfulness.

A hallmark of natural sciences is that scientific hypotheses and scientific theories must make predictions about the natural world (Paz-y-Miño and Spinosa, 2011). Often, the older the theory, the more reliable it is because it has survived many empirical tests. Furthermore, the more universal the theory, the more robust it becomes with time, as more tests would have been performed. According to Darwin, evolutionary theory is centred around two points (Darwin, 1859). First, from one generation to the next, natural populations change over time by a process of natural selection. Second, all organisms have a common ancestor, and the time since this last common ancestor lived is inversely proportional to the similarities that the organisms will share today. Hence, evolutionary theory is universal because it includes all (living and fossil) biological diversity and has implications for all heritable characteristics of life. Since 1859, evolutionary theory has become the most universal and, hence, widely tested of the scientific theories in biology.

Today, Darwin’s original theory has been refined, as he himself anticipated that it would be (Darwin, 1871). This occurred in many fronts because recent concepts, such as genetic drift and mutations, have provided more details on *how* natural populations evolve. One example is the understanding that, at the molecular level, random evolution, rather than natural selection, plays the most important role (Kimura, 1991). This is known as the neutral theory, which completed its 50^th^ anniversary in 2018.

The substance of Darwin’s original theory, however, remains. Theodosius Dobzhansky (1973) shared his astonishment that Charles Darwin proposed the theory of evolution without many key biological concepts, such as that DNA is the molecule responsible for heredity. Half a century after Dobzhansky’s paper, it remains impressive that the theory of evolution still stands valid in light of the discoveries of the molecular biology revolution. Each newly sequenced genome tests some aspects of Darwin’s theory, and, on each case, the sequence has been consistent with Darwin’s prediction of the shared evolutionary history of life. The sharp increase in scope and universality of evolution has strengthened Darwin’s original proposal and made evolutionary theory one of the most reliable and tested theories in the natural sciences (National Academy of Sciences, 2008).

Some creationists dispute this information, claiming that scientists discredit data that go against evolutionary theory. Nonetheless, there is no room for considering worldwide, long-lasting conspiracies in science, as scientific fame and recognition come from the demolition of old theories, not from adherence to them (Atkins, 1995). Indeed, scientists themselves have challenged many aspects of the original Darwinian theory of evolution, such as the importance of neutral evolution, the discovery of epigenetics, the proposal of punctuated equilibrium, etc. When these challenges were first proposed, they were not ignored; they were published in top scientific journals and have been subject to meticulous research and have generated fruitful debates in the scientific arena.

Furthermore, if scientists were dishonestly accepting a false theory of evolution, Lamarck’s theory of inheritance of acquired characters would still be considered valid today. However, it is not. In the XIX century, August Weissman (1889) removed the tails of 20 generations of mice, but no significant decrease in length was found in the descendants’ tails. Scientists themselves devised the scientific experiment that bluntly rejected Lamarck’s proposal as a mechanism of evolution (Dobzhansky, 1973). Scientists do not discredit data that goes against evolution; otherwise, Lamarck’s idea would still be accepted. They discredit scientific untestable theories and explanations that were not gathered using the scientific method.

## The cornerstone of biology

**Figure 2:**
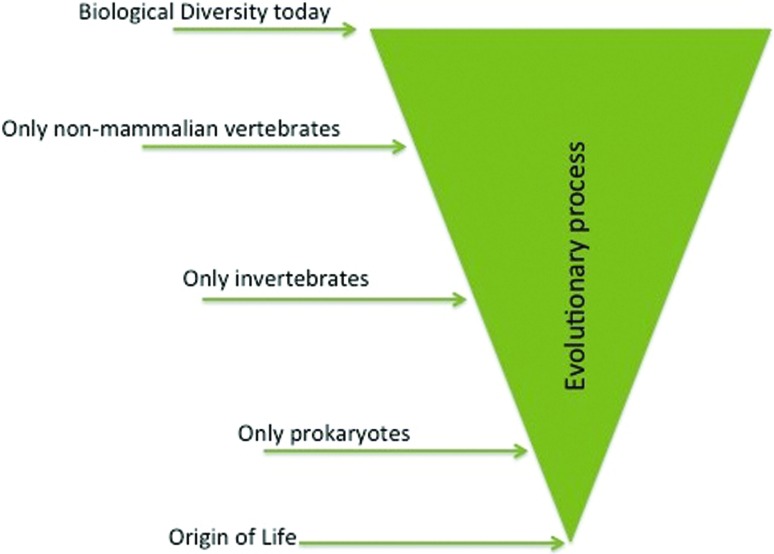
The relationship between evolution and biology. Ever since the origin of life, evolutionary processes, such as mutation, selection, and genetic drift, are responsible for genetic change in natural and laboratory populations. Hence, biology is an instantaneous snap-shot of the dynamic evolutionary axis. This simplified figure illustrates some, of the many, faunal explosions that have took place since the origin of life.

Just as human history explains the geopolitical configurations of our world today, modern biological systems are a direct result of their evolutionary past. Hence, evolutionary theory is the cornerstone of the discipline of biology (Rutledge and Warden, 2000). The discipline of biology today is an instantaneous portrayal of the dynamic evolutionary axis that arose with the origin of life and has been changing by evolution ever since (Figure 2). With the first life, genetics, ecology, biochemistry and evolution began.

As a scientific theory, however, which facts does evolutionary theory explain? One pivotal example is the succession in the fossil record. This evolution, namely, macroevolution, explains the larger evolutionary picture that is the appearance of the greater groups, such as the evolution of mammals, insects, and plants. Fossilized mammals are easily recognized, as they have distinct types of teeth, such as molars, canines, and incisors. These vertebrates are also very likely to fossilize on account of their rigid teeth and hard cranium. If mammals are so easily fossilized, how can we explain a rich fossil record full of vertebrates and invertebrates with no mammalian fossil before 300 million years ago?

Similarly, if we dig deeper still, disclosing 500 million years old layers, we find no hard skeleton vertebrates but plenty of fossilized invertebrates in a boost of diversity that we call the Cambrian Explosion. There are no vertebrates in this explosion because vertebrates appear in a much later explosion. Digging even deeper, to 600 million years old records, we find strata with soft-bodied Ediacaran animals but no hard-shelled invertebrates and no vertebrates. In one billion years old strata, we find only single-celled organisms.

How can we find, in old strata, many single celled organisms but not a single mammalian tooth? The only reasonable explanation for these facts is that 400 million years ago, mammals had not yet evolved; 500 million years ago, vertebrates had not yet evolved; 600 million years ago, hard-shelled invertebrates had not yet evolved; and one billion years ago, multicellular life had not yet evolved. Smaller local successions are also observable in the fossil record; such as the beautiful strings of intermediate fossils that include amphibians (Kustchera and Elliot, 2013), birds, whales [Bibr B32](Thewissen, 2009), horses, and humans. These successions in the fossil record are the most obvious evidence to macroevolution (Figure 2). In fact, the entire fossil record is a set of millions of intermediate fossils that provide solid evidence of how macroevolution worked in the past billion years.

## Evolutionary processes that drive micro and macroevolution are facts

To have a better understanding of evolution, we must discuss the processes that drive evolution. For this, we start by comparing processes that drive microevolution with those that drive macroevolution. Many of the same evolutionary processes that drive microevolution also drive macroevolution, namely natural selection, mutation, migration, and genetic drift. A lineage will tend to diversify if it has adaptations that increase survival and reproductive abilities compared to other species. This advantage will tend to increase population size and the geographical distribution of the ancestral species that will more likely speciate into two descendant species. Hence, according to this view, macroevolution is microevolution on a larger scale (Zimmer, 2001), with biological speciation as the only additional process (Russo *et al.*, 2016). Through speciation, one ancestral species gives rise to two descendant species that are reproductively incompatible with each other.

More than a million species have been described (Mora *et al.*, 2011), and each biological species includes many interbreeding members. Also, most species are reproductively isolated from each other. The fact that we observe biological species with interbreeding members and reproductive isolation between species is compatible with both separate creation and macroevolution. So, which observable pattern would we expect if many speciation events generated the vast biological diversity from a single common ancestor? In this case, we would expect different degrees of similarity between reproductively isolated species. This is exactly what we observe. Some species are very similar, such as chimpanzees and gorillas, with most features shared between them. Other species, on the other hand, are morphologically so different that one must look into cytology, physiology, or comparative genomics to detect evidence of their common past. One example is a fern and a frog. For instance, the cellular respiration is a process shared by ferns and frogs and it is an evidence of their common ancestry. Only macroevolution explains well the distinct degrees of similarity between these four isolated species, as the age of their last common ancestor is inversely proportional to the similarity between any two species.

Furthermore, the existence of hybrids, such as the mule, the liger, the coywolf, is also only explained by the hierarchical common ancestry theory, not by separate creation. The hybrids are direct evidence of on-going processes of speciation. Thus, the presence of hybrids is what we would expect if all life had a common ancestry.

Other fossil record patterns are well explained by macroevolution. For instance, why do we find a major increase in mammalian fossil diversity only after the disappearance of non-avian dinosaurs approximately 65 million years ago? The same pattern is observed in the fossil record of birds. Macroevolution explains this well, as the extinction of dinosaurs eliminated competition, and the surviving ancestral mammals were able to increase in number and diversified through speciation, generating more species of their kind.

## Final remarks

A single, very well designed experiment, performed in accordance with the utmost scientific standards, is what it takes to put any scientific theory to rest. Divine creation will never be part of science because science is not able to detect supernatural phenomena. Divine phenomena explain everything equally; hence, it provides no real explanatory (i.e., predictive) power. If we accept “God’s will” as an adequate explanation for a natural phenomenon, we eliminate the possibility of eventually being able to explain it naturally. Thus, the scientific revolution begun when we eliminated the divine as a scientific explanation.

Science, as a process, starts with the acceptance of our ignorance about a natural phenomenon and by seeking natural explanations for it. Hence, ignorance drives the engine of Science. Even if evolution were, hypothetically, rejected, contested by new data, scientists would have to study hard to find an alternative *natural* explanation that was able to explain everything that evolution explains today plus the new data that contested it.

Evolution is a fact and a well-supported scientific theory. It has endured daily and rigorous testing, and it stands as the unifying theory in biology (Rutledge and Warden, 2000). This says nothing about whether God created or did not create the world, as science is unable to distinguish a divinely guided evolution from a materialistic evolution. God may well have created the biological world through natural selection, mutation, speciation, extinction, etc. Still, evolution and Science would remain unscathed as Science is not concerned with *why* or *who*, but only with *how*.

Some creationists say that we must bring the evolution *versus* creationist debate to the classroom and claim that the opposition to the debate is anti-scientific. However, science is not about blind criticism (Meyer and El-Hani, 2013). Blind criticism is just as naïve as blind acceptance. Scientists must weigh the evidence before questioning a theory. The idea that all debates are equally scientific is misleading and it explains the sad emergence of flat-earthers and anti-vaxxers. A debate on what is the shape of our planet is not only pointless, but it is also dangerously harmful to the minds of the young students. A fruitful debate in a science class is restricted to those issues that lie within the scientific realm (Baltzley, 2016, Branch, 2016).

A recent study has suggested that science concepts, more than evolutionary basics, are critical to promoting evolution (Dunk *et al.*, 2017). One way to reinforce these fundamentals would be the requirement of evolution and science fundaments in admission policies for biology professionals, particularly teachers (Larkin and Perry-Ryder, 2015; see Rutledge and Warden, 2000 for statistics).
